# Supplementary material for: Differential effects of COVID-19 and containment measures on mental health: Evidence from ITA.LI—Italian Lives, the Italian household panel
Source: PLoS One. 2021 Nov 16;16(11):e0259989. doi: 10.1371/journal.pone.0259989 (PMC8594801; doi:10.1371/journal.pone.0259989)
Supplement: S1 File — (DOCX) [file pone.0259989.s001.docx]

**S1 File**

**Figure 1. Kernel distribution of the pre- and post-Covid mental health latent-trait scores**


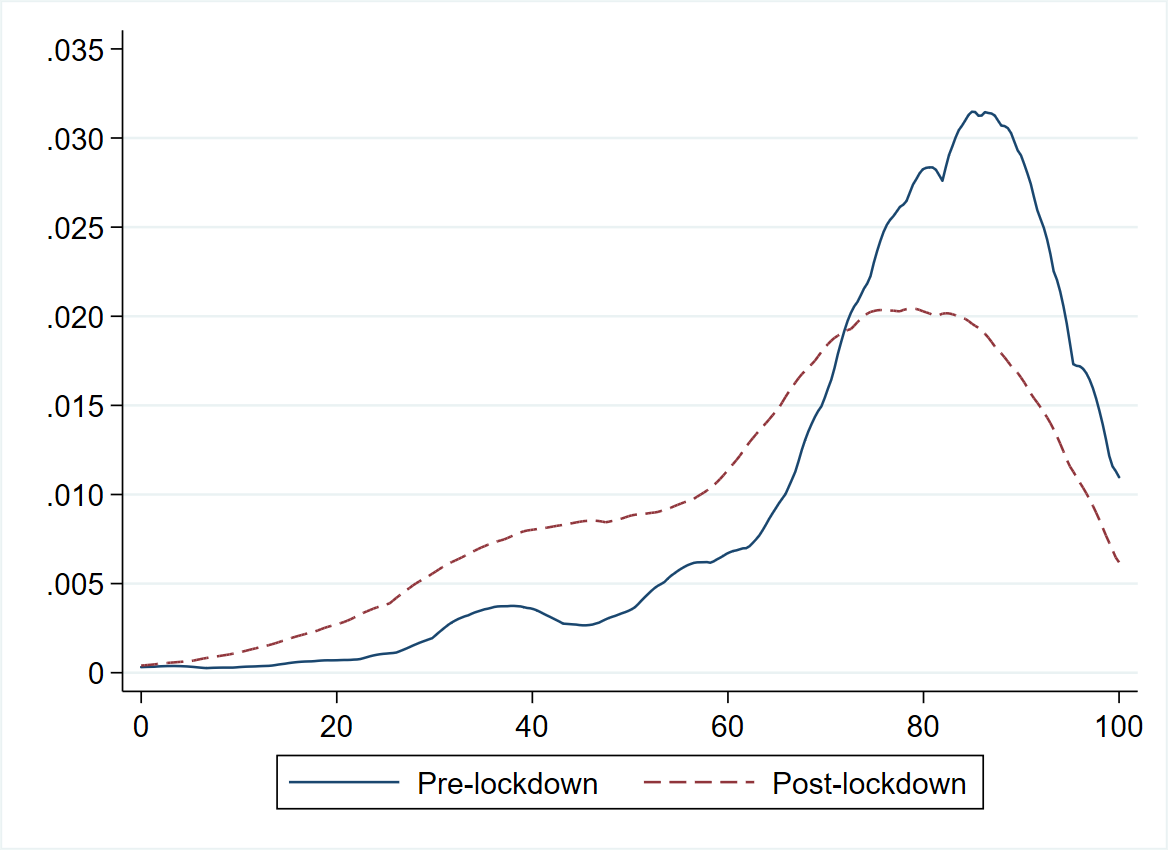


**Table 1. Results of the sensitivity analysis on potential seasonality effects (N=257). Within-person change in mental health associated with the Covid-19 pandemic.**

|  | **Pre-lockdown**  **(95% CI)** | | **Post-lockdown**  **(95% CI)** | | **Pre-Post**  **average change**  **(95% CI)** | | **Cohen’s d**  **(Effect size)** | **p-value** | **Wald test** |
| --- | --- | --- | --- | --- | --- | --- | --- | --- | --- |
|  |  |  |  |  |  |  |  |  |  |
| Total sample | 79.2 | (77.1; 81.3) | 68.9 | (66.4; 71.4) | -10.4 | (-13.0; -7.6) | -0.479 | <0.001 |  |
|  |  |  |  |  |  |  |  |  |  |
| Age |  |  |  |  |  |  |  |  |  |
| 16–34 | 85.4 | (81.0; 89.8) | 68.3 | (60.7; 76.0) | -17.1* | (-24.7; -9.6) | -0.774 | <0.001 | 0.101 |
| 35–44 | 83.8 | (78.1; 84.5) | 69.6 | (62.2; 77.0) | -14.2* | (-22.5; -5.9) | -0.599 | 0.001 |  |
| 45–54 | 81.6 | (77.5; 85.8) | 70.7 | (65.4; 76.1) | -10.9* | (-16.8; -5.0) | -0.514 | <0.001 |  |
| 55–69 | 75.9 | (71.8; 79.9) | 66.9 | (61.7; 72.0) | -9.0* | (-14.5; -3.5) | -0.409 | 0.001 |  |
| 70 or more | 73.2 | (67.2; 79.1) | 70.9 | (63.2; 78.5) | -2.3 | (-10.0; -5.4) | -0.135 | 0.557 |  |
| Living with a partner |  |  |  |  |  |  |  |  |  |
| No | 75.8 | (72.3; 79.3) | 68.4 | (64.2; 72.6) | -7.4* | (-11.8; -3.0) | -0.338 | 0.001 | 0.086 |
| Yes | 82.1 | (79.8; 84.4) | 69.6 | (66.2; 73.0) | -12.5* | (-16.0; -8.9) | -0.319 | <0.001 |  |
| Testing for COVID-19 |  |  |  |  |  |  |  |  |  |
| No | 79.8 | (77.7; 81.9) | 69.0 | (66.4; 71.7) | -10.8* | (-13.7; -7.9) | -0.498 | <0.001 | 0.355 |
| Yes | 76.5 | (71.6; 81.4) | 69.7 | (60.8; 78.5) | -6.8 | (-14.6; -0.9) | -0.305 | 0.084 |  |
| Increase in mortality rate at the municipal level |  |  |  |  |  |  |  |  |  |
| Up to 10% | 78.4 | (75.6; 81.2) | 68.3 | (64.5; 72.1) | -10.1* | (-14.2; -6.0) | -0.461 | <0.001 | 0.960 |
| 11-50% | 78.7 | (75.2; 82.1) | 68.3 | (64.0; 72.6) | -10.3* | (-14.9; -5.7) | -0.440 | <0.001 |  |
| 51% or more | 83.9 | (79.8; 88.0) | 72.7 | (67.0; 78.4) | -11.2* | (-17.1; -5.3) | -0.619 | <0.001 |  |
| Sex |  |  |  |  |  |  |  |  |  |
| Male | 80.8 | (77.7; 83.8) | 71.9 | (68.1; 75.8) | -8.8* | (-12.8; -4.9) | -0.447 | <0.001 | 0.341 |
| Female | 78.5 | (76.0; 81.3) | 67.0 | (63.6; 70.4) | -11.5* | (-15.2; -7.8) | -0.497 | <0.001 |  |
| Age of the youngest child |  |  |  |  |  |  |  |  |  |
| No child aged 0–14 | 79.8 | (77.6; 82.0) | 70.0 | (67.1; 72.9) | -9.8* | (-12.9; -6.7) | -0.467 | <0.001 | 0.261 |
| 0–6 years | 82.9 | (77.9; 87.9) | 65.2 | (57.0; 73.4) | -17.6* | (-27.1; -8.1) | -0.859 | <0.001 |  |
| 7–14 years | 73.8 | (64.7; 82.9) | 66.0 | (56.3; 75.6) | -7.9 | (-18.8; -3.1) | -0.304 | 0.162 |  |
| Education° |  |  |  |  |  |  |  |  |  |
| Up to lower secondary | 78.6 | (75.0; 82.1) | 69.1 | (64.4; 73.8) | -9.5* | (-14.6; -4.3) | -0.426 | <0.001 | 0.436 |
| Upper secondary | 80.6 | (77.7; 83.5) | 70.3 | (66.5; 74.1) | -10.3* | (-14.3; -6.2) | -0.495 | <0.001 |  |
| Tertiary | 78.1 | (73.2; 83.1) | 66.0 | (59.7; 72.4) | -12.1* | (-18.7; -5.5) | -0.582 | <0.001 |  |
| Employment status° |  |  |  |  |  |  |  |  |  |
| Employed | 79.3 | (76.3; 82.2) | 69.8 | (65.7; 73.9) | -9.5* | (-13.7; -5.2) | -0.455 | <0.001 | 0.652 |
| Unemployed | 79.2 | (72.3; 86.0) | 61.3 | (49.5; 73.0) | -17.9 | (-29.4; -6.4) | -0.836 | 0.002 |  |
| Economically inactive | 74.1 | (68.5; 79.7) | 67.0 | (59.2; 74.7) | -7.2 | (-15.7; 1.3) | -0.273 | 0.097 |  |
| Retired | 82.6 | (78.2; 87.0) | 70.2 | (63.7; 76.7) | -12.4* | (-19.1; -5.7) | -0.607 | <0.001 |  |
| Shortage of living space° |  |  |  |  |  |  |  |  |  |
| No | 80.1 | (78.2; 82.0) | 69.5 | (66.8; 72.2) | -10.6* | (-13.4; -7.7) | -0.490 | <0.001 | 0.700 |
| Yes | 74.2 | (65.5; 82.8) | 65.5 | (58.3; 72.8) | -8.6 | (-17.9; 0.6) | -0.352 | 0.068 |  |

° *Lagged variables measured in ITA.LI wave 1*

** Statistically significant at the 5% level after the Bonferroni correction for multiple testing in Model 2.*
