# Supplementary material for: Differential effects of COVID-19 and containment measures on mental health: Evidence from ITA.LI—Italian Lives, the Italian household panel
Source: PLoS One. 2021 Nov 16;16(11):e0259989. doi: 10.1371/journal.pone.0259989 (PMC8594801; doi:10.1371/journal.pone.0259989)
Supplement: Attachment — Submitted filename: Response to reviewers.docx [file pone.0259989.s003.docx]

**Rebuttal letter**

Dear editor and reviewers, please find listed below the responses to each point raised during the review process. Responses are in *italics*.

**Reviewer 1**

- Abstract: The relative and absolute differences are still not clear – it is important to the readers that it is clear if it is a small but significant effect

- It is also important to mention in the abstract that there was no significant changes for the group >70 years and that it was most pronounced among the youngest group 16-34 years of age were there were a 21% absolute decline on the score

*We revised the abstract accordingly*

- It should be highlighted that there might be a bias of the individuals attending here for the second time, which was under the lockdown, and they might be biased towards more likelihood of people responding that felt affected by the pandemic. So this should also be clear with how many in total took part of the first investigation, and how many of these also responded here the second time, also in the abstract. As the attendance seemed to be low for this follow-up investigation, which could be a considerable bias

*We clearly acknowledged this limitation in the final section of the paper. Please note that due to word limit count we could not add all this information in the abstract*

- And good that the authors sorted out the number of participants, which ended up to be different than the two numbers initially reported

*We would like to thank you for your comments and notes to previous round of reviews, which helped in sorting this issue out.*

- I don’t really see how the authors see that hypothesis 2 was really fulfilled, as there are many outcomes in hust this hypothesis, where most where not significant. So if their hypothesis 2 were just if there were any differences in any subgroups, these should also be adjusted for multiple testing regarding all the subgroups included in this

*We revised the discussion of the results accordingly. In particular, building on Kuhn and colleagues we hypothesized (Hypothesis 2) a stronger deterioration of mental health among those more exposed to the detrimental effects of social isolation, such as young adults, individuals living without a partner, and people belonging to a COVID-19 risk group (i.e. those tested because had symptoms or were potentially exposed to the virus and people living in municipalities with high rates of new coronavirus infections) (Hypothesis 2). In the revised version of the study we clearly stated, in a separate paragraph (to make it clearer), as follows:*

*“The findings of the multiple-interaction model revealed that the mental health of the younger age groups was the most severely affected. For those aged 16–34 years, the estimated mental health score was -17.7 points (p < .001, Cohen’s d = -.744) lower than the pre-lockdown baseline measurement. The mental health score was also -14.1 points lower in people aged 35–44 years (p < .001, Cohen’s d = -.621); however, those aged 55–69 years experienced a relatively small reduction of -7.4 points (p < .001, Cohen’s d = -.316). For individuals aged 70+ no statistically significant change in mental health scores was recorded. For individuals living without a partner, those tested because had symptoms or were potentially exposed to the virus, and people living in municipalities with high rates of new coronavirus infections the COVID-19 crisis failed to bring about statistically significant worsening mental health. Hence, the social isolation hypothesis (Hypothesis 2) was confirmed only with respect to age.”*

*Please note that in the previous version of this study we already stated that Hypothesis 2 was only partially confirmed, just with respect to age. Besides, we had already run multiple testing analyses in previous round of review, too.*

- How was hypothesis 3 sustained?

*We revised the discussion of the results accordingly. In particular, again, building on Kuhn and colleagues, as well as on previous research conducted during the pandemic, we hypothesized (Hypothesis 3) a stronger deterioration of mental health among individuals with a heavier workload, such as women and people with preschool-age children in the household (Hypothesis 3). In the revised version of the paper, we discussed the results as follows:*

*“Furthermore, comparisons of estimated marginal means showed that downward trends in mental health scores were significantly more pronounced for women (β = -12.1, p < .001, Cohen’s d = -.504) than for men (β = -8.1, p < .001, Cohen’s d = -.354), and for those cohabiting with and taking care of children aged 0–6 years (β = -16.2, p < .001, Cohen’s d = -.678) than for individuals in households with no children below the age of 14 (β = -10.4, p < .001, Cohen’s d = -.439). Accordingly, the heavier workload hypothesis (Hypothesis 3) was fully sustained.”*

- It might be easier to read if they have subheadings in the result section for each of the hypothesis investigated

*In third-round review, we tested the hypotheses and presented the findings in separate paragraphs, which we believe improved the clarity and readability of the paper.*

- Overall, I'm also concerned how much their used questions actually represent the mental health. Results for specific questions are not presented, and the overall score seem to also have questions as "accomplished less" and "limited social activities", which a lockdown will induce for most, but it doesn't answer how their mental health is affected, and you can score significantly lower on these items for instance, but still have the same overall mental health. Like the question "feeling downhearted and low" is a bit closer to what the article tries to answer, so how is the difference on this question for instance?

*The 12-item Short Form Survey (SF-12) is a general health questionnaire that was first published in the mid-90s as part of the Medical Outcomes Study (MOS). It is one of the most widely used measure of mental health. See, for instance: Ware J, Kosinski M, Keller SD. A 12-item short-form health survey: construction of scales and preliminary tests of reliability and validity. Med Care. 1996; 34:220–233. doi: 10.1097/00005650-199603000-00003. Hence, it has been extensively studied and employed over time. In this study we had already provided additional evidence of the reliability and robustness of the mental health-related scale that includes the selected items. Furthermore, in response to your concerns, please find below some additional analyses, which further support the scale.*

| Variable | Frequencies (%) | | | | | | p-value |
| --- | --- | --- | --- | --- | --- | --- | --- |
| During the past 4 weeks, have you had any of the following problems with your work or other regular daily activities as a result of any emotional problems (such as feeling depressed or anxious)?  Have you accomplished less than you would like? | | | | | | | |
|  | Yes | No |  |  |  |  |  |
| Pre | 93 (11) | 765 (89) | - | - | - | - | <0.001* |
| Post | 250 (29) | 608 (71) | - | - | - | - |  |
| Didn't do work or other activities as carefully as usual? | | | | | | | |
|  | Yes | No |  |  |  |  |  |
| Pre | 86 (10) | 772 (90) | - | - | - | - | <0.001* |
| Post | 246 (29) | 612 (71) | - | - | - | - |  |
| These questions are about how you feel and how things have been with you during the past 4 weeks. For each question, please give the answer that comes closest to the way you have been feeling. How much of the time during the past 4 weeks…  Have you felt downhearted and low? | | | | | | | |
|  | All of the time | Most of the time | A good bit of the time | Some of the time | A little of the time | None of the time |  |
| Pre | 7 (1) | 29 (3) | 29 (3) | 163 (19) | 422 (49) | 208 (24) | <0.001^¥^ |
| Post | 18 (2) | 54 (6) | 49 (6) | 261 (30) | 321 (37) | 155 (18) |  |
| Did you have a lot of energy? | | | | | | | |
|  | None of the time | A little of the time | Some of the time | A good bit of the time | Most of the time | All of the time |  |
| Pre | 9 (1) | 48 (6) | 218 (25) | 186 (22) | 280 (33) | 117 (14) | 0.006^¥^ |
| Post | 12 (1) | 89 (10) | 262 (31) | 90 (11) | 304 (35) | 101 (12) |  |
| Have you felt calm and peaceful? | | | | | | | |
|  | None of the time | A little of the time | Some of the time | A good bit of the time | Most of the time | All of the time |  |
| Pre | 8 (1) | 36 (4) | 162 (19) | 183 (21) | 329 (38) | 140 (16) | 0.001^¥^ |
| Post | 10 (1) | 63 (7) | 227 (27) | 101 (12) | 344 (40) | 113 (13) |  |
| During the past 4 weeks, how much of the time has your physical health or emotional problems interfered with your social activities? | | | | | | | |
|  | All of the time | Most of the time | Some of the time | A little of the time | None of the time |  |  |
| Pre | 6 (1) | 29 (3) | 100 (12) | 202 (24) | 521 (61) | - | <0.001^¥^ |
| Post | 17 (2) | 60 (7) | 215 (25) | 311 (36) | 255 (29) | - |  |
|  |  |  |  |  |  |  |  |
| * p-values derived from the Mc Nemar’s test for dichotomous variables  ^¥^ p-values derived from the Wilcoxon signed rank sum test for ordered categorical variables | | | | | | | |

Reviewer #2: Dear Authors, Thank you for your careful consideration of my comments. I am overall happy with your replies to my comments. I have some very minor comments:

-The abbreviation RE and FE are not introduced in the paper

*We revised the text accordingly*

-In table 3 in the last column, it should be mentioned that this is the p-value of the Wald test

*We revised the text accordingly*
